# Butterfly Pea Flower (*Clitoria ternatea* Linn.) Extract Ameliorates Cardiovascular Dysfunction and Oxidative Stress in Nitric Oxide-Deficient Hypertensive Rats

**DOI:** 10.3390/antiox10040523

**Published:** 2021-03-27

**Authors:** Putcharawipa Maneesai, Metee Iampanichakul, Nisita Chaihongsa, Anuson Poasakate, Prapassorn Potue, Siwayu Rattanakanokchai, Sarawoot Bunbupha, Petcharat Chiangsaen, Poungrat Pakdeechote

**Affiliations:** 1 Department of Physiology, Faculty of Medicine, Khon Kaen University, Khon Kaen 40002, Thailand; putcma@kku.ac.th (P.M.); metee.iam@kkumail.com (M.I.); nisita@kkumail.com (N.C.); anuson_p@kkumail.com (A.P.); prappo@kku.ac.th (P.P.); 2 Faculty of Veterinary Medicine, Khon Kaen University, Khon Kaen 40002, Thailand; siwara@kku.ac.th; 3 Faculty of Medicine, Mahasarakham University, Maha Sarakham 44000, Thailand; sarawoot.b@msu.ac.th; 4 Faculty of Medicine, Bangkokthonburi University, Bangkok 10170, Thailand; petcharat.chi@bkkthon.ac.th; 5 Research Institute for Human High Performance and Health Promotion, Khon Kaen University, Khon Kaen 40002, Thailand

**Keywords:** *Clitoria ternatea* Linn., left ventricular and vascular dysfunction, oxidative stress, renin–angiotensin system, inflammation

## Abstract

In this study, we examine whether *Clitoria ternatea* Linn. (CT) can prevent Nω-nitro-L-arginine methyl ester hydrochloride (L-NAME)-induced cardiac and vascular dysfunction in rats. Male Sprague Dawley rats were given L-NAME (40 mg/kg, drinking water) and orally administered with CT extract (300 mg/kg/day) or lisinopril (2.5 mg/kg/day) for 5 weeks. The main phytochemical components of the CT extract were found to be flavonoids. The CT extract alleviated the high blood pressure in rats receiving L-NAME. Decreased vasorelaxation responses to acetylcholine and enhanced contractile responses to sympathetic nerve stimulation in aortic rings and mesenteric vascular beds of L-NAME treated rats were ameliorated by CT extract supplementation. Left ventricular hypertrophy and dysfunction were developed in L-NAME rats, which were partially prevented by CT extract treatment. The CT extract alleviated upregulated endothelial nitric oxide synthase expression, decreased plasma nitrate/nitrite levels, and increased oxidative stress in L-NAME rats. It suppressed high levels of serum angiotensin-converting enzyme activity, plasma angiotensin II, and cardiac angiotensin II type 1 receptor, NADPH oxidases 2, nuclear factor-kappa B, and tumor necrosis factor-alpha expression. The CT extract, therefore, partially prevented L-NAME-induced hypertension and cardiovascular alterations in rats. These effects might be related to a reduction in the oxidative stress and renin–angiotensin system activation due to L-NAME in rats.

## 1. Introduction

Since nitric oxide (NO) was discovered more than 40 years ago, it has been well-established as playing a crucial physiological role in blood pressure control [1]. A reduction in NO causes an impairment of endothelial function, which has been implicated in the pathogenesis of hypertension [2]. In animal models of hypertension, the blockade of NO synthesis using nitric oxide synthase inhibitors to induce cardiovascular alterations has been well-accepted [3,4]. Chronic inhibition of NO production by Nω-nitro-L-arginine methyl ester (L-NAME) produces high blood pressure, cardiovascular hypertrophy, and dysfunction in rats. These deleterious effects of L-NAME are associated with NO depletion, renin–angiotensin system (RAS) activation, and reactive oxygen species (ROS) over-production [5,6]. Modulation of the RAS has been suggested to participate in the development of hypertension in L-NAME-treated rats, as increased angiotensin-converting enzyme (ACE) activity has been observed in serum [7] and tissue [8] in this animal model. Angiotensin II (Ang II) is a potent vasoconstricting peptide produced by the proteolytic cleavage of angiotensin I by ACE in endothelial cells. Ang II binds to its receptors, angiotensin II type 1 receptor (AT_1_R), to mediate vasoconstriction, inflammation, hypertension, and cardiac dysfunction [9].

Oxidative stress has been implicated as closely related to the pathogenesis of hypertension. Many studies have shown high levels of systemic lipid peroxidation and low levels of endogenous antioxidant enzymes in hypertensive patients and animals [10,11]. Additionally, there exists evidence that increased oxidative stress markers in hypertensive patients are suppressed by AT_1_R antagonists [12]. There is growing evidence that Ang II can induce ROS production by the activation of the AT_1_R/NADPH oxidase signaling cascade in hypertension [13,14,15,16]. Oxidative stress is one of possible mechanisms which might contribute to L-NAME-induced hypertension in animal models. In L-NAME-treated rats, the link between RAS activation and ROS production has been evidenced. For example, high levels of plasma Ang II, the upregulation of AT_1_R and NADPH oxidase subunits (e.g., p47^phox^ and gp91^phox^) together with increases in vascular O_2_^•−^ and plasma malondialdehyde (MDA) levels, as well as a reduction in NO metabolites (NOx), have been described in L-NAME-induced hypertensive rats [5,6,17,18]. Subsequently, eNOS uncoupling has been demonstrated to be a major source of superoxide generations in L-NAME hypertensive rats [19,20]. Low levels of endogenous antioxidant enzymes, such as superoxide dismutase, catalase, and glutathione, have been noted in NO-deficient hypertensive rats [21].

Cardiac dysfunction and hypertrophy are the consequent complications of high blood pressure. These cardiac abnormalities are related to the adaptive response to pressure load and several molecular pathways. It has been reported that the activation of the nuclear factor-kappa B (NF-κB) signaling pathway is required for the hypertrophic growth of cardiomyocytes [22]. NF-κB is an oxidative stress-sensitive transcriptional factor for inflammation, immune response, and cell growth; its activity is stimulated by several hypertrophic agonists, such as endothelin-1 and Ang II [22,23,24]. Additionally, the upregulation of NF-κB—together with the excessive expression of pro-inflammatory cytokines such as interleukin-6 (IL-6) and tumor necrosis factor-alpha (TNF-α)—associated with cardiac hypertrophy has been observed in L-NAME hypertensive rats [25]. Additionally, L-NAME hypertensive rats have demonstrated changes in cardiac morphology and function [26]. Therefore, reducing RAS over-activation, oxidative stress, and inflammation might have beneficial effects for the prevention of cardiovascular alterations associated with hypertension.

Butterfly pea (or Blue-pea) is a common name of *Clitoria ternatea* Linn. (CT), which is a plant species in the Fabaceae family, widely distributed in Asia, Africa, and Australia. This plant has been gained more interest in recent years, as it has exhibited antioxidant, anti-inflammatory, antidiabetic, hypolipidemic, anticancer, and anti-platelet-aggregation properties [27,28,29,30,31,32]. It includes several phytochemical compounds, such as tannins, anthocyanins, flavonoid glycosides, triterpenoids, saponins, tannins, phenols, anthraquinone, and cardiac glycosides. [33,34]. A recent study has reported that the consumption of CT (as a beverage) increased plasma antioxidant capacities without hypoglycemia in healthy subjects [35]. However, little information regarding the protective effects of CT extracts on nitric oxide (NO) depletion-induced cardiovascular abnormalities has been demonstrated. The present study was designed to evaluate the preventive effects of CT extract on cardiac and vascular dysfunction in rats receiving L-NAME. We also explored the effects of CT extract on oxidative stress, the activation of the renin–angiotensin system and inflammation in L-NAME rats.

## 2. Materials and Methods

### 2.1. Preparation of CT Flower Extract and Chemical Analysis of the Extract

Dry CT flowers were obtained from the Vejpong Pharmacy (Hock An Tang) company limited (Vejpong Pharmacy, Co., Ltd., Bangkok, Thailand) and were extracted in boiling water for 1 h. Thereafter, the water extract was filtered and freeze-dried to CT powder using a lyophilizer (Labconbo, Becthai Bangkok Equipment & Chemical Co., Ltd.). The yield (calculation on the dried powder extract) was 27.78% w/w of the dried CT. The CT powder was packed into containers and kept at −20 °C until further use. To analyze the phytochemical components of the extract, reverse-phase ultra-high performance liquid chromatography coupled with quadrupole time-of-flight mass spectrometry (RP-UHPLC-QTOF-MS) was applied.

### 2.2. Animals and Induction of Hypertension

Male Sprague Dawley rats weighing 220–250 g were purchased from Nomura Siam International Co, Ltd., Bangkok, Thailand. Rats were housed in an HVAC (Heating, Ventilation, and Air-Conditioning) system (23 ± 2 °C) with a 12 h dark/light cycle at the Northeast Laboratory Animal Centre. All animal procedures complied with the standards for the care and use of experimental animals and were approved by the Animal Ethics Committee of Khon Kaen University, Khon Kaen, Thailand (AEKKU-NELAC 72/2561). 

The animals were randomly divided into 4 groups (*n* = 8/each group), as follows: the control group received vehicle (drinking water, 1.5 mL/kg, p.o.), the L-NAME group received L-NAME and vehicle (1.5 mL/kg, p.o.), and the L-NAME-treated group received L-NAME and either CT extract (300 mg/kg/day, p.o.) or lisinopril (2.5 mg/kg/day, p.o.). L-NAME (40 mg/kg/day) was dissolved in drinking water for 5 weeks in order to induce hypertension in the L-NAME group, while control rats were given tap water.

### 2.3. Indirect Measurement of Blood Pressure in Conscious Rats

Indirect blood pressure was measured weekly throughout the 5 weeks of the study using non-invasive tail cuff plethysmography (IITC/Life Science Instrument model 229 and model 179 amplifier; Woodland Hills, CA, USA). Conscious rats were placed on a restrainer and allowed to acclimate prior to blood pressure measurement. The measurement was repeated three times and expressed as mean values from each rat.

### 2.4. Cardiac Function Study

At the end of the experiment, rats were anesthetized with thiopental sodium (70 mg/kg, i.p.) and echocardiogram was performed using a Model LOGIQ S7 (GE Healthcare, WI, USA). Left ventricular (LV) structure and function were assessed from two-dimensional short-axis view and M-mode tracings were recorded for the LV internal dimension at the end-diastole (LVIDd), end-systole (LVIDs), interventricular septum at diastole (IVSd) and systole (IVSs), LV posterior wall thickness at diastole (LVPWd) and at systole (LVPWs), end diastolic volume (EDV), end systolic volume (ESV), and stroke volume (SV) from three consecutive cardiac cycles. LV fractional shortening (% FS) was calculated by the following equation: % LVFS = [(LVIDd − LVIDs) / LVIDd] × 100.

### 2.5. Direct Measurement of Blood Pressure in Rats under Anaesthesia

After cardiac function measurement, the left femoral artery was identified and cannulated using a polyethylene tube. Baseline values of systolic blood pressure (SBP), diastolic blood pressure (DBP), mean arterial pressure (MAP), and heart rate (HR) were continuously monitored for 20 min by way of a pressure transducer and recorded using the Acknowledge Data Acquisition software (Biopac Systems Inc., Santa Barbara, CA, USA).

### 2.6. Vascular Function Study

After hemodynamic assessment, the mesenteric vascular bed and the thoracic aorta were carefully isolated, then paced on a stainless-steel grid (7 × 5 cm^2^) in a humid chamber [36]. To deplete sensory neurotransmitters and desensitize vanilloid receptors, the mesenteric beds were perfused with physiological Krebs solution containing capsaicin (0.1 μM) for 20 min, followed by 30 min for the washout period. The contractile responses to electrical field stimulation (EFS; at 5–40 Hz, 90 V, 1 ms, for 30 s at 5-min intervals) or exogenous norepinephrine (NE) (0.15–15 nmol) were performed. Moreover, to determine the vasoactive performance of resistant small arteries, ACh (0.1 nM to 0.1 mM) or sodium nitroprusside (SNP, 0.1 nM to 0.1 mM) was injected through neoprene rubber tubing proximal to the tissue under rising tone with methoxamine (5–7 μM). Changes in mean perfusion pressure (mmHg) were detected using a pressure transducer and the data were recorded using the BIOPAC System (BIOPAC Systems Inc., Santa Barbara, CA, USA). In another set of experiments, the thoracic aorta was cut into rings, mounted in 15 mL baths containing Krebs solution at 37 °C, and gassed with a 95% O_2_ and 5% CO_2_ gas mixture. Isometric contractions were recorded with a resting tension of 1 g using a transducer connected to a 4-channel bridge amplifier, a PowerLab A/ D converter, and a PC running the Chart v5 software (PowerLab System, ADInstruments, Australia). ACh (0.001–3 μM)-induced endothelial-mediated relaxations and vascular response to SNP (0.001–3 μM) were assessed by pre-contracting with phenylephrine (10 μM); relaxation is expressed as % relaxation.

### 2.7. Assay of Oxidative Stress Markers

Blood samples were drawn and collected into ethylenediaminetetraacetic acid (EDTA) collection tubes. The ventricular tissue was homogenized with phosphate lysis buffer and the supernatant was obtained. The levels of malondialdehyde (MDA) in plasma and ventricular supernatant were measured using thiobarbituric acid-reactive substances (TBARS), following a previously described method [6]. Briefly, 150 µL of the sample was reacted with 10% TCA, 125 µL of 5 mM EDTA, 125 µL of 8% SDS and 10 µL of 0.5 µL/mL of butylated hydroxytoluene (BHT). The mixture was left for 10 min and then 0.6% TBA was added in an equal volume and the mixture was heated for 30 min in a boiling water bath. After cooling to room temperature, the mixture was centrifuged 10,000 g for 5 min at 25 °C. The absorbance of the supernatant was measured at the wavelength of 532 nm by spectrophotometer. A standard curve was generated using appropriate concentrations of standard 1,1,3,3-tetraethoxypropane (TEP) (0.3–10 mmol/L). Vascular superoxide (O_2_^•−^) production in the carotid artery was determined using a chemiluminescence-based technique described previously, with some modifications [17]. In brief, the vessel was incubated with 1 mL oxygenated Krebs-KCl solution at pH 7.4, 37 °C for 30 min. Thereafter, lucigenin 100 mM was added in sample tube and place in a luminometer (Turner Biosystems, Sunnyvale, CA, USA). Luminometer count was integrated every 30 s for 5 min and averaged. Vascular tissue O_2_^•−^ production was expressed as relative light unit count per minute per dried weight of vascular tissues.

### 2.8. Assay of Plasma Nitrate/nitrite (NOx) and TNF-α Level in Cardiac Tissue

The concentrations of plasma NOx levels—the end products of nitric oxide (NO) metabolism—were determined by conversion of nitrate to nitrite using nitrate reductase and followed by Griess reagents, as previously noted [17]. The level of cardiac TNF-α was measured using an enzyme-immunoassay (ELISA) kit (Abcam, Cambridge, MA, USA), following the manufacturer’s instructions.

### 2.9. Assay of Catalase (CAT), Angiotensin-Converting Enzyme (ACE) Activity, and Angiotensin II Level

The level of catalase (CAT) activity was measured in plasma, following a previously described method with some modifications [37]. Briefly, 20 µL of plasma sample was added to a 96-well plate. Then, the sample was mixed with 100 µL of substrate (65 µmol/mL hydrogen peroxide; H_2_O_2_) in 0.06 M sodium-potassium phosphate (HKNaO_4_P) buffer (pH 7.4, at 37 °C for 1 min). The enzymatic reaction was stopped by adding 100 µL of 32.4 mM ammonium molybdate NH_46_(Mo_7_O_24_·4H_2_O). The yellow complex was determined under a 405 nm wavelength. A standard curve was generated with concentrations of bovine liver catalase from 3.125 to 100 U/mL. The value of serum CAT activity is expressed as U/mL. 

Serum ACE activity was determined using a fluorescence assay, as previously described, with some modifications [38]. The serum was mixed with hippuryl-l-histidyl-l-leucine (HHL) in assay buffer, then incubated at 37 °C for 30 min. After that, NaOH was added to stop the reaction and the product of the reaction was fluorogenically labelled with O-phthaldialdehyde (OPA). The fluorescence was read at 355 nm excitation, 535 nm emission using a fluorescent plate reader. Plasma Ang II level was measured using an Angiotensin II EIA Kit (RAB0010; Sigma-Aldrich), as per the standard procedures mentioned in the kit.

### 2.10. Western Blot Analysis of eNOS and AT_1_ Receptor (AT_1_R) and NF-κB Protein Expression

The levels of eNOS, AT_1_R, and NF-κB protein expression in LV tissue were determined using the Western blot method. A mouse monoclonal antibody to eNOS (Cat. 610296, BD Bioscience, San Jose, CA, USA), rabbit polyclonal antibody to AT_1_R (N-10) (sc-1173, Santa Cruz Biotechnology, Inc., Santa Cruz, CA, USA), rabbit polyclonal immunoglobulin G (IgG) to p-NF-κB p65 (S536, Cell Signaling Technology, Inc., Danvers, MA, USA), and mouse monoclonal IgG to gp91^phox^ (sc-74514, Santa Cruz Biotechnology, Inc., Santa Cruz, CA, USA) were used in the current study. β-actin was used as a protein loading control in order to compare the intensities of protein expression.

### 2.11. Statistical Analysis

The results of this study are expressed as mean ± SEM. Statistical analysis was carried out using one-way ANOVA follow by Tukey’s post hoc tests for comparison between groups. A probability value less than 0.05 was considered statistically significant.

## 3. Results

### 3.1. The Main Phytochemical Components in CT Extract

A total of 2110 compounds were detected in the positive electrospray ionization mode and 282 compounds were found in the negative electrospray ionization mode. Background signals were removed when the coefficient of variation was less than 30%. Thereafter, metabolite identification was performed. The main compounds in the aqueous extracts of CT were flavonoids, such as kaempferol 3-glucoside, quercetin 3-rhamnosyl-rhamnosyl-glucoside, rutin, quercetin 3-glucoside, and kaempferol 3-isorhamninoside (see Table 1). In addition, L-tryptophan, an amino acid, was also found in the CT extract.

### 3.2. CT Extract Exhibited an Antihypertensive Effect in L-NAME Hypertensive Rats

**Figure 1:**
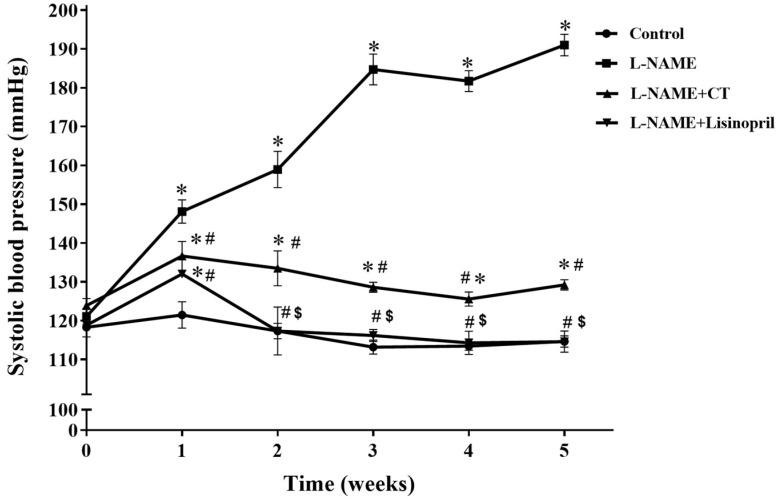
Effects of CT extract and lisinopril on systolic blood pressure in conscious rats. Data are expressed as mean ± SEM (*n* = 8/group), * *p* < 0.05 vs. control, ^#^ *p* < 0.05 vs. L-NAME, ^$^ *p* < 0.05 vs. L-NAME + CT. CT:; *Clitoria ternatea* Linn. extract.

During the five weeks of L-NAME treatment, SBP progressively increased compared with the control group (191 ± 2.98 vs. 114.65 ± 1.48 mmHg; *p* < 0.05; Figure 1). Daily oral administration of CT extract or lisinopril significantly prevented the development of hypertension induced by L-NAME (129.21 ± 1.33 and 118.5 ± 0.80 mmHg, respectively) when compared with untreated hypertensive rats (*p* < 0.05). The dose (300 mg/kg/day) of CT extract used in the present study was based on the result from a preliminary study. CT extract at doses of 100 and 300 mg/kg/day produced dose-dependent anti-hypertensive effects; however, there was no significant difference between 300 and 500 mg/kg/day doses (*n* = 4).

The SBP, DBP, MAP, and HR in anesthetized rats of all groups in the experiment are shown in Table 2. Significant increases in all hemodynamic parameters were observed in L-NAME hypertensive rats compared to the control rats (*p* < 0.05). Treatment with CT extract significantly prevented all hemodynamic alterations caused by L-NAME (*p* < 0.05). Moreover, lisinopril treatment also abolished the hypertension induced by L-NAME, as there were no significant differences of SBP, DBP, MAP, and HR in L-NAME treated with lisinopril and control rats (*p* < 0.05; Table 2).

In addition, heart weight per body weight (HW/BW) and left ventricular weight per body weight (LV/BW) in L-NAME hypertensive rats were significantly increased compared to the control rats (*p* < 0.05). CT extract supplementation significantly reduced HW/BW and LV/BW, as shown in Table 2. Similar results were observed with lisinopril supplementation (*p* < 0.05).

### 3.3. CT Extract Alleviated Vascular Dysfunction in L-NAME Hypertensive Rats

**Figure 2:**
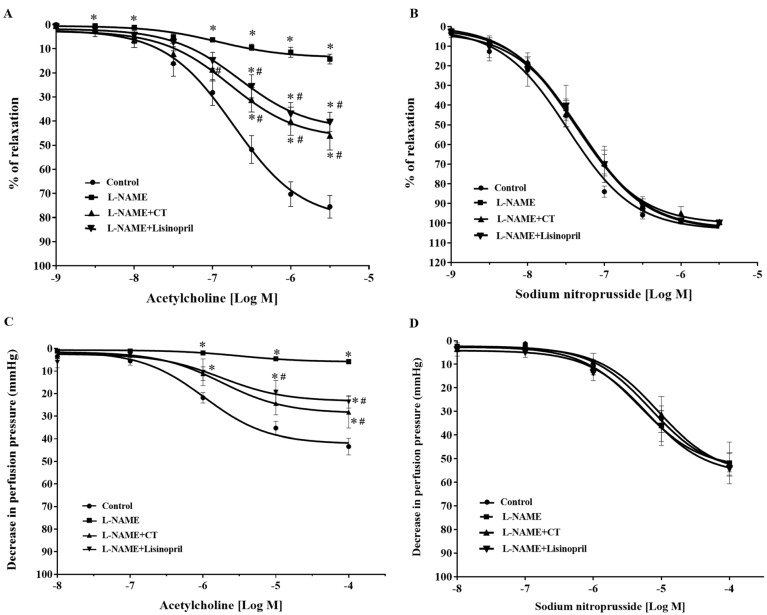
Effects of CT extract and lisinopril on vascular function in thoracic aorta (**A,B**) and mesenteric vascular beds (**C,D**) in all groups of the experiment. Data are expressed as mean ± SEM (*n* = 8/group), * *p* < 0.05 vs. control, ^#^ *p* < 0.05 vs. L-NAME, CT: *Clitoria ternatea* Linn. extract.

After five weeks of treatment, vasorelaxation responses to ACh (0.001–3 μM) were significantly impaired in aortic rings of L-NAME-induced hypertensive rats compared to the response in control rats (at 3 μM ACh: 14.25 ± 2 vs. 75.48 ± 4.67%, *p* < 0.05). Treatment with CT extract or lisinopril significantly improved vasorelaxation responses to ACh (at 3 μM ACh: 45.96 ± 5.97 and 40.34 ± 3.95%, respectively) compared to L-NAME-induced hypertensive rats (*p* < 0.05; Figure 2A). However, vasorelaxation responses to SNP, an NO donor (0.001–3 μM), did not significantly differ among groups (*p* > 0.05; Figure 2B).

In addition, an impairment of endothelial function was confirmed in the mesenteric vascular bed, as significant reductions in vasorelaxation responses to ACh (0.1 nM to 0.1 mM) were observed in the mesenteric vascular beds isolated from L-NAME-induced hypertensive rats compared to the responses in control rats (at 0.1 mM ACh: 9.58 ± 3.9 vs. 37.42 ± 4.71 mmHg, *p* < 0.05). The response was significantly improved in rats treated with CT extract or lisinopril (at 0.1 mM ACh: 24.52 ± 6.96 and 23.65 ± 2.72 mmHg, respectively) compared to the L-NAME hypertensive rats (*p* < 0.05; Figure 2C). However, the vasorelaxation response to SNP (0.1 nM to 0.1 mM) did not significantly differ among groups (*p* > 0.05), which indicated normal vascular smooth muscle cell function (Figure 2D).

### 3.4. CT Extract Attenuated Contractile Responses to Electrical Filed Stimulation (EFS) in Mesenteric Vascular Beds Isolated from L-NAME Hypertensive Rats

**Figure 3:**
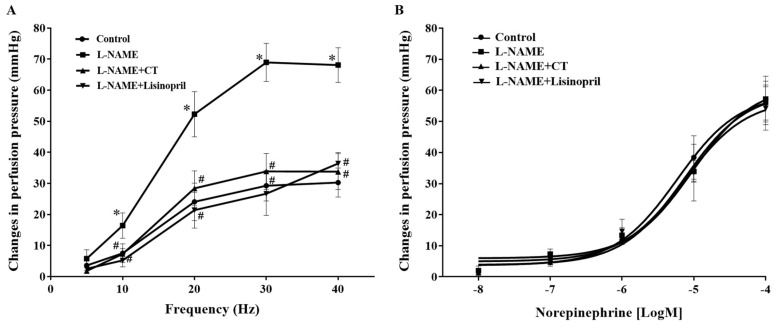
Effects of CT extract and lisinopril on contractile responses to electrical field stimulation (**A**) and exogenous norepinephrine (**B**) in mesenteric vascular beds collected from all groups in the experiment. Data are expressed as mean ± SEM (*n* = 8/group), * *p* < 0.05 vs. control, ^#^ *p* < 0.05 vs. L-NAME. CT: *Clitoria ternatea* Linn. extract.

Frequency-dependent contractile responses to EFS (at 5–40 Hz) were observed in all groups of rats, as shown in Figure 3A. Interestingly, significant enhancements of contractile responses to EFS were observed in the mesenteric vascular beds isolated from L-NAME rats compared to the responses in the control rats (at 40 Hz: 68.09 ± 5.60 vs. 30.26 ± 4.67 mmHg, *p* < 0.05). CT extract or lisinopril treatment attenuated these responses to EFS (33.77 ± 5.79 and 36.38 ± 3.52 mmHg, respectively) compared to the response in untreated rats (*p* < 0.05; Figure 3A). However, the contractile response to exogenous NE (0.15–15 nmol) did not show a significant difference among groups (*p* > 0.05; Figure 3B).

### 3.5. CT Extract Prevented Cardiac Dysfunction Induced by L-NAME in Rats

**Figure 4:**
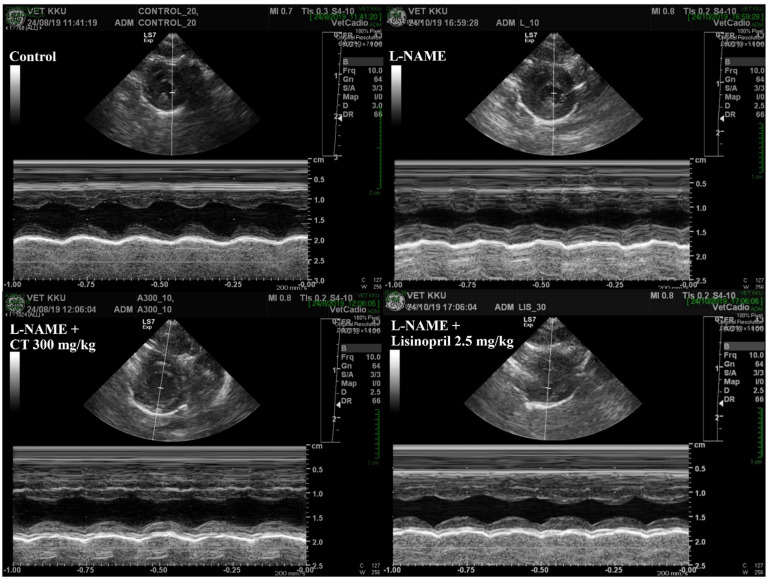
Representative tracings of transthoracic echocardiographs in all groups of rats. CT: *Clitoria ternatea* Linn. extract.

An impairment of LV function was observed in L-NAME hypertensive rats, which was characterized by increases in IVSd and LVPWd and decreases in LVIDd, EDV, %EF, SV, and %FS compared with those of control rats (*p* < 0.05, Table 3). L-NAME rats treated with CT extract showed an improvement in LV function, supported by restorations of IVSd, %EF, SV, %FS, IVSd, and LVPWd compared with untreated rats (*p* < 0.05, Figure 4). In addition, lisinopril significantly reduced IVSd and LVPWd, accompanied with significant improvements in %EF, SV, and %FS compared with untreated L-NAME rats (*p* < 0.05; Table 3).

### 3.6. CT Extract Improved Plasma Nitric Oxide Metabolites (NOx) and Cardiac eNOS Protein Expression in L-NAME Hypertensive Rats

**Figure 5:**
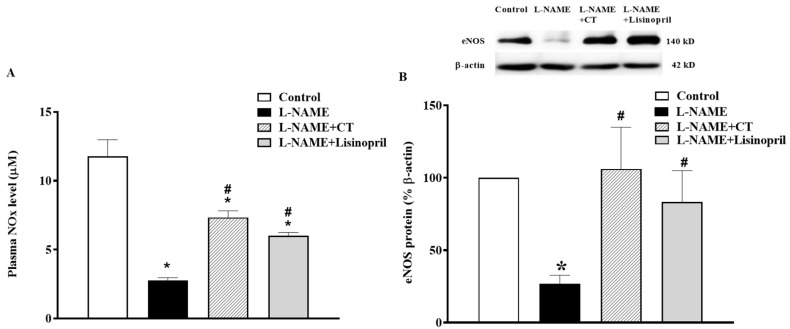
Effects of CT extract and lisinopril on nitric oxide metabolites (**A**) and endothelial nitric oxide synthase (eNOS) protein expressions (**B**) in cardiac tissue of all groups in the experiment. Data are expressed as mean ± SEM (*n* = 4/group), * *p* < 0.05 vs. control, ^#^ *p* < 0.05 vs. L-NAME, CT: *Clitoria ternatea* Linn. extract.

In L-NAME treated rats, a significant decrease in plasma NOx concentration was found compared to the control rats (2.61 ± 0.12 vs. 11.78 ± 1.19 µM, *p* < 0.05). This low level of NOx was restored by CT extract and lisinopril supplementation (7.33 ± 0.48 and 6.02 ± 0.21 µM, respectively; *p* < 0.05; Figure 5A). The NOx level was in accordance with a downregulation of eNOS protein expression in cardiac tissue in L-NAME hypertensive rats compared to the control (Figure 5B). However, treatment with CT extract or lisinopril significantly upregulated eNOS protein expression in L-NAME hypertensive rats compared to untreated rats (*p* < 0.05; Figure 5B).

### 3.7. CT Extract Reduced Oxidative Stress in L-NAME Hypertensive Rats

**Figure 6:**
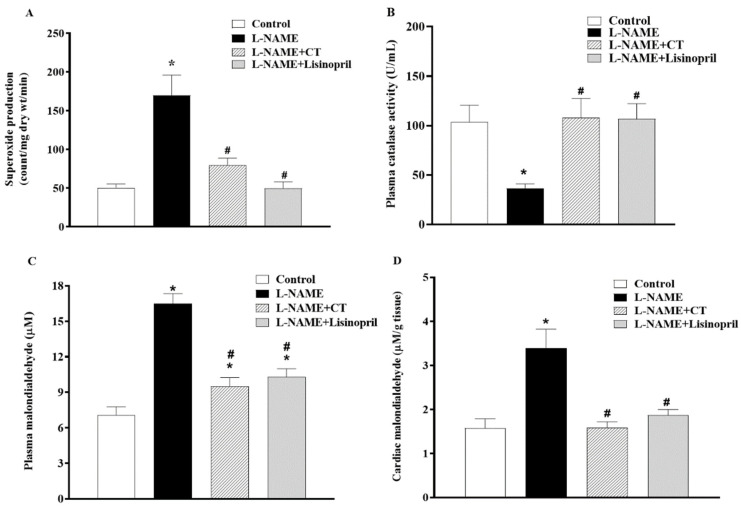
Effects of CT extract and lisinopril on vascular superoxide production (**A**), plasma catalase activity (**B**), plasma malondialdehyde (MDA) (**C**), and cardiac MDA levels (**D**) in all groups in the experiment. Data are expressed as mean ± SEM (*n* = 8/group), * *p* < 0.05 vs. control, ^#^ *p* < 0.05 vs. L-NAME; L: L-NAME, CT: *Clitoria ternatea* Linn. extract.

A high level of vascular O_2_^•−^ production (151.94 ± 7.14 vs. 57.97 ± 3.55 count/mg dry wt/min; *p* < 0.05) and a low level of plasma catalase activity (36.77 ± 4.20 vs. 103.52 ± 17.13 U/mL; Figure 6A,B) were observed in L-NAME hypertensive rats compared to control rats. CT extract or lisinopril treatment alleviated the production of vascular O_2_^•−^ (90.04 ± 3.25 and 84.72 ± 8.72 count/mg dry wt/min, respectively) and restored serum CAT activity back to the normal level (107.9 ± 19.72 and 106.78 ± 15.42 U/mL, respectively), as shown in Figure 6A and B. Moreover, levels of plasma and cardiac MDA were higher in L-NAME rats than those in control rats (16.48 ± 0.86 vs. 7.08 ± 0.68 μM and 3.39 ± 0.43 vs. 1.58 ± 0.21 μM/ g tissue, *p* < 0.05). Supplementation with CT extract and lisinopril restored the level of plasma (9.51 ± 0.73 and 10.30 ± 0.67 μM) and cardiac MDA (1.58 ± 0.13 and 1.88 ± 0.12 μM/ g tissue) in hypertensive rats compared to the untreated group (Figure 6C,D).

### 3.8. CT Extract Suppressed Serum Angiotensin-Converting Enzyme (ACE) Activity, Plasma Angiotensin II (Ang II) Level, Angiotensin II Receptor Type 1 (AT_1_R), NOX2, and p-NF-κB Protein Expressions in Cardiac Tissue Collected from L-NAME Hypertensive Rats

**Figure 7:**
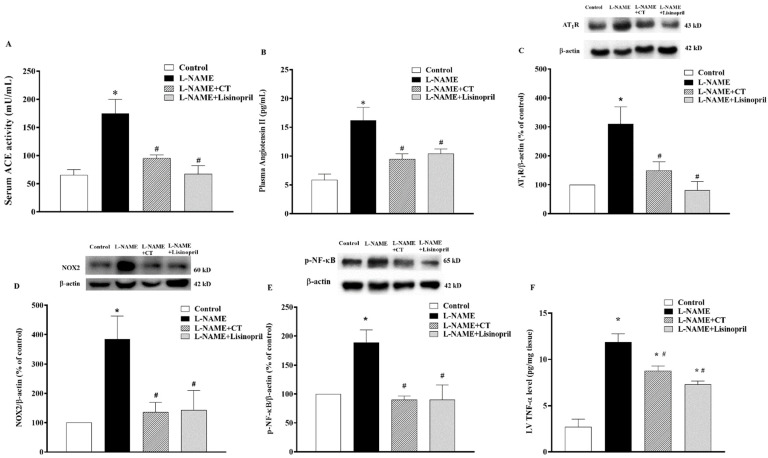
Effects of CT extract and lisinopril on serum angiotensin-converting enzyme activity (ACE, (**A**)), plasma angiotensin II level (Ang II, (**B**)), angiotensin II receptor type 1 (AT_1_R) (**C**), NOX2 (**D**), p-NF-κB protein expressions (**E**), and tumor necrosis factor-alpha (TNF-α) level in cardiac tissue (**F**) of all groups in the experiment. * *p* < 0.05 vs. control, ^#^ *p* < 0.05 vs. L-NAME, CT: *Clitoria ternatea* Linn. extract.

There were significant increases in serum ACE activity and plasma Ang II levels in L-NAME-induced hypertensive rats compared to control rats (174.64 ± 25.07 vs. 65.47 ± 9.40 mU/mL and 16.22 ± 1.01 vs. 5.85 ± 2.20 pg/mL, respectively; *p* < 0.05). CT extract or lisinopril significantly reduced serum ACE activity (95.27 ± 5.78 and 67.61 ± 14.61 mU/mL, respectively) and plasma Ang II levels (9.50 ± 0.87 and 10.41 ± 0.79 pg/mL, respectively) compared to untreated L-NAME hypertensive rats (*p* < 0.05;Figure 7A,B). Interestingly, the overexpression of AT_1_R and NOX2 protein expressions in LV was observed in the L-NAME hypertensive group compared to the control group (*p* < 0.05;Figure 7C,D). CT extract and lisinopril attenuated the overexpression of these proteins, as shown in Figure 7C,D. High levels of TNF-α were found in hypertensive rats, which was relevant to the overexpression of p-NF-κB protein compared to the control group (*p* < 0.05). However, CT extract and lisinopril treatment restored these high levels of TNF-α and p-NF-κB protein expression, as shown in Figure 7E,F.

## 4. Discussion

We found that the main phytochemical components of CT extract are flavonoids. CT extract prevented hypertension induced by NO depletion in rats. The impairment of left ventricular and vascular function in rats receiving L-NAME was protected by treatment with CT extract. Likewise, it increased eNOS protein expression and plasma NOx in L-NAME rats. Oxidative stress, as evidenced by increases in superoxide generation and plasma lipid peroxidation, as well as a reduction in plasma CAT activity in hypertensive rats, was alleviated by CT extract treatment. RAS activation, inflammation, and upregulation of AT_1_R, NOX2, and p-NF-κB in cardiac tissue were observed in L-NAME rats, but were suppressed under CT extract supplementation.

Several lines of evidence have reported that L-NAME-induced hypertension is related to reduce NO bioavailability, resulting in endothelial dysfunction and hypertension [39,40]. This study demonstrated that hypertensive rats had a reduction in vasorelaxant responses to ACh without changes in their response to a NO donor. An enhancement in contractile responses to nerve stimulation was observed in L-NAME rats, while the response to exogenous NE was not altered. These findings were supported by the downregulation of eNOS and low levels of plasma NOx in L-NAME rats. The reduction in eNOS protein expression in the present study was consistent with previous studies [18,41,42]; however, Pechanova and co-workers found increases in eNOS expression and activity in rats treated with L-NAME [43]. The controversy of eNOS expression induced by L-NAME in rats might be caused by different strains of rats, treatment periods and doses of L-NAME, etc. There is evidence to support that NO can modulate sympathetic nerve-mediated contractile responses by deactivating the vasoconstrictor norepinephrine [44]. Moreover, L-NAME treatment can produce ROS by oxidizing tetrahydrobiopterin (the cofactor of NO synthesis), leading to the uncoupling of the NOS dimer. Uncoupled NOS causes a reduction in NOS activity, contributing to producing the O_2_^•−^ radical, rather than NO [45]. This was consistent with our results, in which L-NAME-treated rats showed an increase in oxidative stress determined by increased vascular O_2_^•−^ production, plasma and cardiac MDA levels, and low plasma catalase activity. Another possible mechanism of decreased NO bioavailability in L-NAME rats is that superoxide can rapidly react with NO to form peroxynitrite, a strong oxidant [46]. In this study, only catalase was measured based on the previous reports that the decrease in the steady state of superoxide dismutase (SOD) and catalase was not different in response to oxidative stress conditions [47,48]. Similar reactivities to singlet oxygen for both enzymes were confirmed [49]. Additionally, catalase can be reduced dramatically in the presence of superoxide radicals [50]. Therefore, the measurement of catalase, an intracellular antioxidant enzyme, might imply oxidative stress in this animal model. L-NAME rats receiving CT extract had lower blood pressure than untreated rats. Additionally, vascular dysfunction in L-NAME rats was resolved by CT extract supplementation. This was accompanied by a restoration of eNOS expression and plasma NOx in hypertensive rats. The results demonstrated that the phytochemical components of the CT extract were mainly comprised of flavonoids, including kaempferol 3-glucoside, quercetin 3-rhamnosyl-rhamnosyl-glucoside, rutin, quercetin 3-glucoside, and kaempferol 3-isorhamninoside. It is possible that these flavonoids exerted biological activities to scavenge oxidative stress and, subsequently, raise NO bioavailability. The CT extract possesses antioxidant capacity through its DPPH radical scavenging activity, total polyphenolic and flavonoid contents, and ferric reducing antioxidant power [51]. There is substantial evidence showing that flavonoids have beneficial effects on lowering blood pressure by reducing endothelial cell oxidative stress and increasing NO bioavailability [52]. The present study showed an alleviation of oxidative damage in CT extract-treated rats mediated by reduced vascular O_2_^•−^ production and (plasma and tissue) MDA levels, resulting in increased NO bioavailability, as well as increased eNOS protein expression, in LV tissue.

RAS activation might be one of the possible mechanisms increasing blood pressure and oxidative stress in L-NAME rats. L-NAME hypertensive rats showed high levels of ACE activity, plasma Ang II, and upregulation of AT_1_R protein expression. These results are supported by the fact that renal ACE producing Ang II is necessary for L-NAME-induced hypertension [53]. Furthermore, Ang II-induced oxidative stress through binding on AT_1_R and the subsequent activation of NADPH oxidase subunits to generate O_2_^•−^ has been described [13] in L-NAME-induced hypertension. We found an overexpression of NOX2 in cardiac tissue, which might be responsible for the production of reactive oxygen species in these hypertensive rats. CT extract treatment suppressed RAS activation by reducing serum ACE activity and Ang II generation in L-NAME-treated rats. Such RAS inhibition might contribute to its blood pressure lowering and vasoprotective effects. Our results were supported by another study, in which the inhibitory effects of CT petal extract on ACE activity were observed to mediate its antihypertensive effect [54].

LV dysfunction and hypertrophy were found in rats under long-term exposure to high blood pressure induced by L-NAME. The LV hypertrophy was supported by increased LVW/BW ratio, LVIDd, and LVPWd, while LV dysfunction was characterized by decreasing SV, %EF, and %FS. The impairment of LV contraction in L-NAME rats in the present study was consistent with the results of a previous study that rats receiving L-NAME had cardiac morphology and dysfunction, as evidenced by increases in IVSd, IVSs, and LVWd, as well as decreases in LVIDd, EDV, %EF, SV, and %FS [5]. These cardiac disruptions induced by L-NAME were alleviated in rats treated with CT extract. This might suggest that the cardioprotective effects of CT extract in the present study are likely relevant to its beneficial effects on blood pressure, vascular function, oxidative stress, and RAS activation. Additionally, the molecular mechanisms involved in the effect of CT extract on cardiac abnormalities were revealed, as associated with inflammation through the Ang II/AT_1_R/NOX2/NF-κB pathway. We found that the elevated NF-κB expression and TNF-α in the cardiac tissue of hypertensive rats were suppressed in CT extract-treated rats. Several studies have described that the activation of the NF-κB signaling pathway is required for the hypertrophic growth of cardiomyocytes [22,55]. Ang II-induced ROS production leading to the activation of NF-κB and inflammation has been well-described [16]. Furthermore, the preventive effects of CT extract on lipopolysaccharide-induced inflammation in macrophage cells have been shown [56].

Lisinopril has been widely recommended for hypertension treatment. It reduces blood pressure through the inhibition of ACE activity [57]. Our results showed that lisinopril prevented L-NAME-induced hypertension, improved LV and vascular function, and suppressed oxidative stress and the Ang II/AT_1_R/NOX2/NF-κB pathway. In addition to reducing ACE activity, lisinopril has been shown to possess other beneficial effects, such as antioxidation, anti-inflammation, and cardiovascular protection [37,58,59,60].

## 5. Conclusions

In conclusion, CT extract and lisinopril prevented the L-NAME-induced development of hypertension and, therefore, were associated with the alleviation of cardiovascular dysfunction in rats. These effects were associated with the suppression of RAS activation, oxidative stress, and inflammation due to the modulation of the AT_1_R/NOX2/NF-κB pathway.

## Figures and Tables

**Table 1:** Main compounds in CT extract, as assayed by reverse-phase ultra-high performance liquid chromatography coupled with quadru-pole time-of-flight mass spectrometry.

RT	[M + H]^+^ (*m/z*)	Identified Compounds	Formula	Class of Phytochemicals
7.38	449.11	Kaempferol 3-glucoside	C_21_H_20_O_11_	Flavonoids
6.84	757.22	Quercetin 3-rhamnosyl-rhamnosyl-glucoside	C_33_H_40_O_20_	Flavonoids
7.28	611.16	Rutin	C_27_H_30_O_16_	Flavonoids
7.46	465.10	Quercetin 3-glucoside	C_21_H_20_O_12_	Flavonoids
7.39	741.22	Kaempferol 3-isorhamninoside	C_33_H_40_O_19_	Flavonoids

RT, retention time; *m/z*, mass-to-charge ratio; CT, *Clitoria ternatea* Linn.

**Table 2:** Effects of CT extract and lisinopril on blood pressure, heart rate, heart weight per body weight, and left ventricular weight per body weight.

Parameters	Control	L-NAME	L-NAME + CT (300 mg/kg)	L-NAME + Lisinopril (2.5 mg/kg)
**SBP (mmHg)**	117.63 ± 5.11	187.39 ± 4.56 *	130.95 ± 2.82 * ^#^	131.18 ± 4.59 * ^#^
**DBP (mmHg)**	72.64 ± 6.01	133.29 ± 4.65 *	86.07 ± 2.31 * ^#^	96.31 ± 4.43 * ^#^
**MAP (mmHg)**	87.64 ± 5.37	151.32 ± 4.55 *	101.03 ± 2.94 * ^#^	109.55 ± 4.67 * ^#^
**HR (beat/min)**	331.74 ± 24.13	383.08 ± 12.56 *	332.02 ± 6.55 ^#^	333.33 ± 15.65 ^#^
**HW/BW (g)**	0.264 ± 0.006	0.272 ± 0.005 *	0.270 ± 0.006 ^#^	0.271 ± 0.005 ^#^
**LV/BW (g)**	0.171 ± 0.002	0.187 ± 0.004 *	0.169 ± 0.003 ^#^	0.174 ± 0.002 ^#^

Data are expressed as mean ± SEM. *n* = 8/group, * *p* < 0.05 vs. control, # *p* < 0.05 vs. L-NAME. SBP: systolic blood pressure, DBP: diastolic blood pressure, MAP: mean arterial pressure, HR: heart rate, HW/BW: heart weight per body weight, LV/BW: left ventricular weight per body weight, CT: *Clitoria ternatea* Linn. extract.

**Table 3:** Effects of CT extract and lisinopril on ventricular dysfunction in all groups of the experiment.

Parameters	Control	L-NAME	L-NAME + CT (300 mg/kg)	L-NAME + Lisinopril (5 mg/kg)
**IVSd (mm)**	1.77 ± 0.03	2.59 ± 0.30 *	1.65 ± 0.08 ^#^	1.63 ± 0.11 ^#^
**IVSs (mm)**	2.68 ± 0.12	3.26 ± 0.43	2.74 ± 0.17	2.65 ± 0.14
**LVIDd (mm)**	6.62 ± 0.27	4.91 ± 0.36 *	6.60 ± 0.37 ^#^	5.70 ± 0.44
**LVIDs (mm)**	3.71 ± 0.17	3.41 ± 0.34	4.00 ± 0.28	3.32 ± 0.28
**LVPWd (mm)**	2.08 ± 0.07	2.81 ± 0.22 *	1.94 ± 0.06 ^#^	2.19 ± 0.19 ^#^
**LVPWs (mm)**	2.78 ± 0.12	3.14 ± 0.27	2.80 ± 0.08	2.70 ± 0.36
**EDV (mL)**	0.68 ± 0.08	0.30 ± 0.07 *	0.67 ± 0.09 ^#^	0.55 ± 0.06
**ESV (mL)**	0.14 ± 0.02	0.12 ± 0.04	0.17 ± 0.03	0.15 ± 0.02
**EF (%)**	79.27 ± 2.86	66.33 ± 4.51 *	76.39 ± 1.40 ^#^	78.66 ± 2.16 ^#^
**SV (mL)**	0.55 ± 0.08	0.19 ± 0.04 *	0.50 ± 0.06 ^#^	0.43 ± 0.04 ^#^
**FS (%)**	43.78 ± 2.78	30.95 ± 3.22 *	40.90 ± 1.08 ^#^	42.08 ± 2.10 ^#^

Data are expressed as means ± SEM. (*n* = 5–8/group). * *p* < 0.05 vs. control, ^#^ *p* < 0.05 vs. L-NAME. IVSd: interventricular septum thickness at end diastole, IVSs: interventricular septum thickness at end systole, LVIDd: left ventricular internal dimension at end-diastole, LVIDs: left ventricular internal dimension at end-systole, LVPWd: left ventricular posterior wall thickness in diastole, LVPWs: left ventricular posterior wall thickness in systole, EDV: end-diastolic volume, ESV: end systolic volume, EF: ejection fraction, SV: stroke volume, FS: fractional shortening, CT: *Clitoria ternatea* Linn. extract.

## Data Availability

No new data were created or analyzed in this study.
